# A Novel Prognostic Model of Early-Stage Lung Adenocarcinoma Integrating Methylation and Immune Biomarkers

**DOI:** 10.3389/fgene.2020.634634

**Published:** 2021-01-21

**Authors:** Jin Ren, Yun Yang, Chuanyin Li, Lu Xie, Ronggui Hu, Xiong Qin, Menghuan Zhang

**Affiliations:** ^1^School of Medicine, Guizhou University, Guiyang, China; ^2^Cancer Center, Shanghai Tenth People’s Hospital, School of Medicine, Tongji University, Shanghai, China; ^3^Shanghai Center for Bioinformation Technology, Shanghai Academy of Science and Technology, Shanghai, China; ^4^State Key Laboratory of Molecular Biology, CAS Center for Excellence in Molecular Cell Science, Chinese Academy of Sciences, Shanghai, China; ^5^Department of Thoracic Surgery, Shanghai Pulmonary Hospital, School of Medicine, Tongji University, Shanghai, China

**Keywords:** lung adenocarcinoma, methylation, CD8 T cell, prognostic model, survival analysis

## Abstract

Lung adenocarcinoma (LUAD) is caused by multiple biological factors. Therefore, it will be more meaningful to study the prognosis from the perspective of omics integration. Given the significance of epigenetic modification and immunity in tumorigenesis and development, we tried to combine aberrant methylation and tumor infiltration CD8 T cell-related genes to build a prognostic model, to explore the key biomarkers of early-stage LUAD. On the basis of RNA-seq and methylation microarray data downloaded from The Cancer Genome Atlas (TCGA), differentially expressed genes and aberrant methylated genes were calculated with “DEseq2” and “ChAMP” packages, respectively. A Chi-square test was performed to obtain methylation driver genes. Weighted correlation network analysis (WGCNA) was utilized to mine cancer biomarkers related to CD8 T cells. With the consequences of univariate Cox proportional hazards analysis and least absolute shrinkage and selection operator (LASSO) COX regression analysis, the prognostic index based on 17 methylation driver genes (ZNF677, FAM83A, TRIM58, CLDN6, NKD1, NFE2L3, FKBP5, ITGA5, ASCL2, SLC24A4, WNT3A, TMEM171, PTPRH, ITPKB, ITGA2, SLC6A17, and CCDC81) and four CD8 T cell-related genes (SPDL1, E2F7, TK1, and TYMS) was successfully established, which could make valuable predictions for the survival risk of patients with early-stage LUAD.

## Introduction

Lung cancer accounts for a large proportion of tumor-related deaths, with 1.7 million deaths worldwide annually (Muller et al., 2019), which can be classified into SCLC and NSCLC. The most common subtype of NSCLC is LUAD (Herbst et al., 2008). Although early diagnosis and treatment techniques have improved significantly in the past few decades, the 5-year OS rate of LUAD patients is still less than 15% (Yang et al., 2019). Moreover, there was no OS benefit for most pathological stage I patients who were received adjuvant therapy (Pignon et al., 2008; Strauss et al., 2008).Therefore, there is an imminent need to seek more accurate predictors from patients with early-stage LUAD to distinguish high-risk subgroups, which can benefit from personalized therapy.

Accumulating evidence suggests that the progression of LUAD is controlled not only by the inherent genetic changes of cancer cells but also by epigenetic and environmental factors, such as abnormal methylation. DNA methylation is a key element of epigenetic modifications and plays an important role in regulating cellular functions and carcinogenesis (Bernstein et al., 2007; Zheng et al., 2017). Many LUAD prognostic models have been established using methylation data, and a series of methylation-related biomarkers have been discovered (Kuo et al., 2016; Li et al., 2019). CD8 T cell is also a factor. Cancer-infiltrating CD8 T cells have a vital effect on the immune response in lung cancer (Hiraoka et al., 2006; Nakanishi et al., 2009; Bos and Sherman, 2010). The outcome of tumor development, as well as the responsiveness to cancer immunotherapy, can be influenced by the total number of T cells found within a tumor. Hence, several immune-related prognostic models have been developed (Li et al., 2017; Zhang et al., 2020). In short, previous prognostic models of LUAD only focused on a single biological factor. Given the significance of epigenetic modification and immunity in tumorigenesis and development, we tried to combine aberrant methylation and tumor infiltration CD8 T cells-related genes to build a prognostic model, and to explore the key biomarkers of early-stage LUAD.

## Materials and Methods

### Data Retrieving and Analyzing

Methylation and mRNA microarray data were retrieved from TCGA (Tomczak et al., 2015). Our study focused on 363 samples, including 345 cancer tissues and 18 normal tissues, which have corresponding DNA methylation, mRNA expression, and complete clinical follow-up information. These samples are early-stage LUAD, including tumor stage I and II. GSE72094 dataset from GEO was used as the validation data, which contained 321 stage I and stage II LUAD patients.

### Candidate Methylation Driver Gene Selection

Differentially expressed mRNAs between cancer and normal samples were identified with “DEseq2” package in R (Love et al., 2014). Genes with | FC| ≥ 1.5 and adjusted *P* ≤ 0.05 were identified as differentially expressed. Aberrant methylated genes were calculated with “ChAMP” package (Tian et al., 2017). Genes with FC ≥ 0.25 and adjusted *P* ≤ 10^–5^ were identified as aberrant methylated. The correlation between differentially expressed genes and aberrantly methylated genes was calculated using Pearson method. Genes with correlation coefficient ≤ −0.3 and adjusted *P* ≤ 0.05 were labeled as methylation driver genes.

To further explore the functions of these methylation driver genes, GO and KEGG enrichment analysis were performed with the “clusterProfiler” package in R (Yu et al., 2012).

### Candidate CD8 T Cell-Related Gene Selection

CD8 T cell is extremely important for immune defense against intracellular pathogens and tumor surveillance. The patient survival and response to immunotherapy can be predicted by tumor-infiltrating CD8 T cells in many cancer types (Pagès et al., 2005; Galon et al., 2006; Azimi et al., 2012; Herbst et al., 2014; Tumeh et al., 2014; Eroglu et al., 2018; Peranzoni et al., 2018; Savas et al., 2018). The total proportion of 22 kinds of immune cells was obtained with CIBERSORT analysis (Newman et al., 2015). Based on transcriptome profiling data and CIBERSORT immune fractions, we employed the WGCNA algorithm (Langfelder and Horvath, 2008) to identify the co-expression module that was most correlated with CD8 T cells.

### Construction of Risk Assessment Signature

Here, we combined methylation driver genes and CD8 T cell-related genes as candidate omics genes. First, univariate Cox proportional hazards regression analysis was conducted to initially screen for genes that were significantly related to OS (*P* ≤ 0.05). Then, LASSO Cox regression model was established with “glmnet” package (Friedman et al., 2010). Finally, genes with non-zero beta values were identified as potential prognosis biomarkers. Risk score was calculated by the following formula:

$$\mathrm{Risk}\mathrm{score}=\sum_{i=1}^{N}Coefficient\left({\mathrm{Gene}}_{i}\right)\times Expression\left({\mathrm{Gene}}_{i}\right)$$

The expression value of each gene was normalized by log2. Specifically, risk assessment signature was established based on 17 methylation driver genes (ZNF677, FAM83A, TRIM58, CLDN6, NKD1, NFE2L3, FKBP5, ITGA5, ASCL2, SLC24A4, WNT3A, TMEM171, PTPRH, ITPKB, ITGA2, SLC6A17, and CCDC81) and four CD8 T cell-related genes (SPDL1, E2F7, TK1, and TYMS).

Next, patients were sorted into high-risk (*n* = 173) and low-risk groups (*n* = 174) with the median risk score as the cutoff. Kaplan–Meier analysis and log-rank tests were used to compare the difference in prognostic time. The ROC curve was adopted to evaluate the predictive capability of the risk assessment signature with the “ROCR” package in R (Sing et al., 2005).

### Construction of Prognostic Nomogram

A genomic-clinical nomogram was established to quantitatively predict the survival probability of each patient. The prediction ability of the nomogram was measured by AUC. The AUC value is between 0.5 and 1.0, and a larger AUC indicates better performance. A calibration curve was derived by comparing the predicted value of the nomogram with the observed survival rates of 3 and 5 years. The prognostic nomogram and calibration curve were generated with the “rms” package (Harrell, 2016). The time-dependent ROC curve was produced with “timeROC” package (Blanche et al., 2013).

### The Further Prognostic Value of Candidate Omics Genes

To further evaluate the prognostic value of these candidate omics genes, gene expression value was used as grouping indicator to perform survival analysis. The median gene expression was designed as a cut-off value for comparing difference in survival rate. *P* ≤ 0.05 was regarded as statistically significant. Kaplan–Meier plot was conducted.

## Results

### Clinicopathological Characteristics of Patients

**FIGURE 1:**
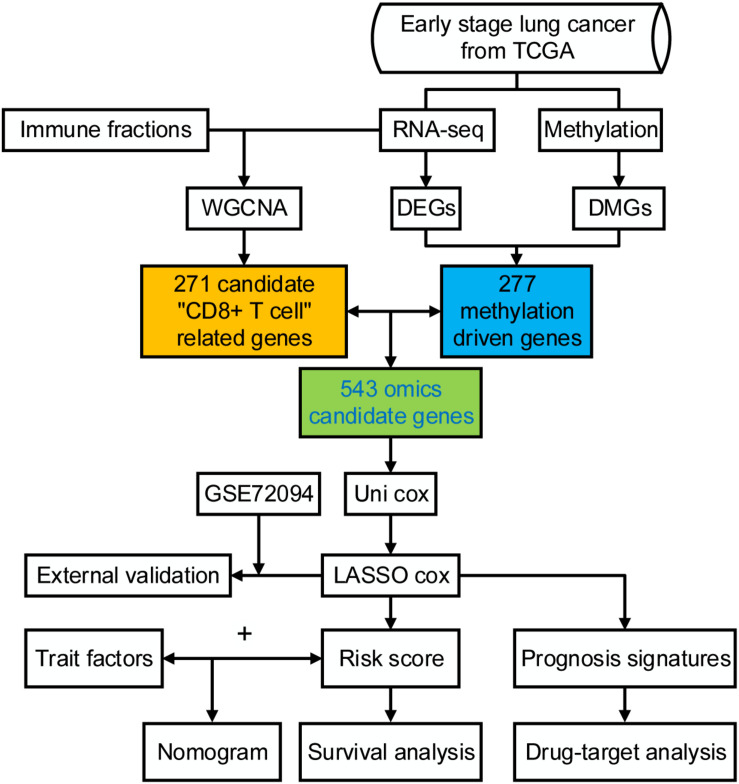
The workflow of the study.

The clinical information of 877 samples was acquired from TCGA. We selected patients with tumor stage I and II. A total of 345 patients with complete clinical follow-up information, mRNA, and methylation microarray data were eventually retained as further study objects. The clinicopathological characteristics are shown in Table 1. The study flowchart is shown in Figure 1.

**TABLE 1:** Clinicopathologic characteristics of early-stage LUAD patients.

	Total (*N* = 345)
Female	188 (54.5%)
Male	157 (45.5%)
**Age (years)**	
Mean (SD)	65.4 (9.95)
Median (Min, Max)	66.0 (33.0, 88.0)
Missing	9 (2.6%)
**Vital_status**	
Alive	240 (69.6%)
Dead	105 (30.4%)
**Smoking**	
No	9 (2.6%)
Yes	336 (97.4%)
**Race**	
Asian	4 (1.2%)
Black or African American	38 (11.0%)
Not reported	29 (8.4%)
**Stage**	
Stage i	5 (1.4%)
Stage ia	115 (33.3%)
Stage ib	118 (34.2%)
Stage ii	1 (0.3%)
Stage iia	49 (14.2%)
Stage iib	57 (16.5%)

### Potential Methylation Driver Genes

**FIGURE 2:**
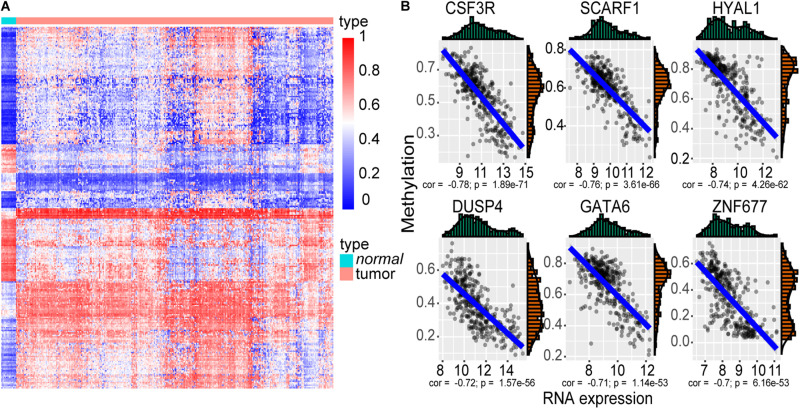
The methylation driver genes. **(A)** Heatmap of the 277 methylation driver genes. The color from blue to red indicates the trend from hypomethylation to hypermethylation. **(B)** Scatter plot of the top six negatively correlated genes. The *x*-axis represents the RNA-seq expression value, and the *y*-axis represents the methylation beta value.

We identified 4501 up-expressed and 2688 down-expressed genes in cancer tissues compared to normal tissues. A total of 2672 aberrant methylated genes were observed, including 1529 hypermethylation and 1143 hypomethylation genes. Then, 277 methylation driver genes were acquired (Figure 2A and Supplementary Table S1), whose mRNA expression was significantly negatively correlated with their corresponding methylation levels. That is, 193 genes with hypermethylation may lead to decreased mRNA expression level and 84 genes with hypomethylation may lead to increased mRNA expression level. The top six negatively correlated genes are shown in Figure 2B, including CSF3R, SCARF1, HYAL1, DUSP4, GATA6, and ZNF677.

**FIGURE 3:**
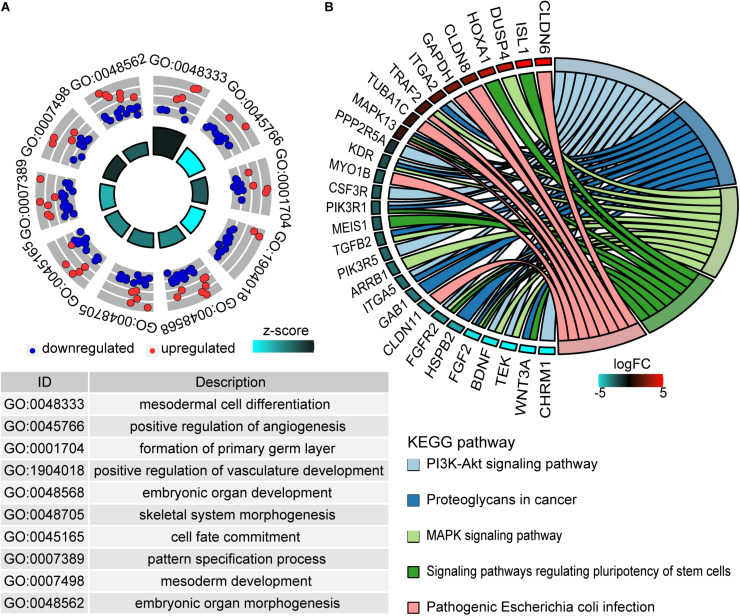
Functional enrichment analysis of methylation driver genes. **(A)** The top 10 enriched terms of the biological process of GO. The outer circle represents the expression values (log2FC) of methylation driver genes in each enriched GO term. Red dots imply upregulated genes and blue dots imply downregulated genes. The inner circle indicates the significance of GO terms. **(B)** The chord plot of top five KEGG enriched pathways. The left outer semicircle represents the log2FC value of methylation driver genes, and the right semicircle corresponds to five pathway entries.

Gene Ontology analysis deciphered that 277 genes were enriched in 199 GO biological process terms (adjust *P* ≤ 0.05) (Supplementary Table S2). The top 10 biological process terms are shown in Figure 3A. These genes were mainly concentrated in mesodermal cell differentiation, positive regulation of angiogenesis, embryonic organ development, cell fate commitment, and so on. Additionally, 25 pathways were significantly associated with these genes (*P* < 0.05) according to KEGG pathway analysis (Supplementary Table S3). The most enriched pathways were PI3K-Akt signaling pathway, proteoglycans in cancer, MAPK signaling pathway, signaling pathways regulating pluripotency of stem cells, and so on (Figure 3B).

### Candidate CD8 T Cell-Related Genes

**FIGURE 4:**
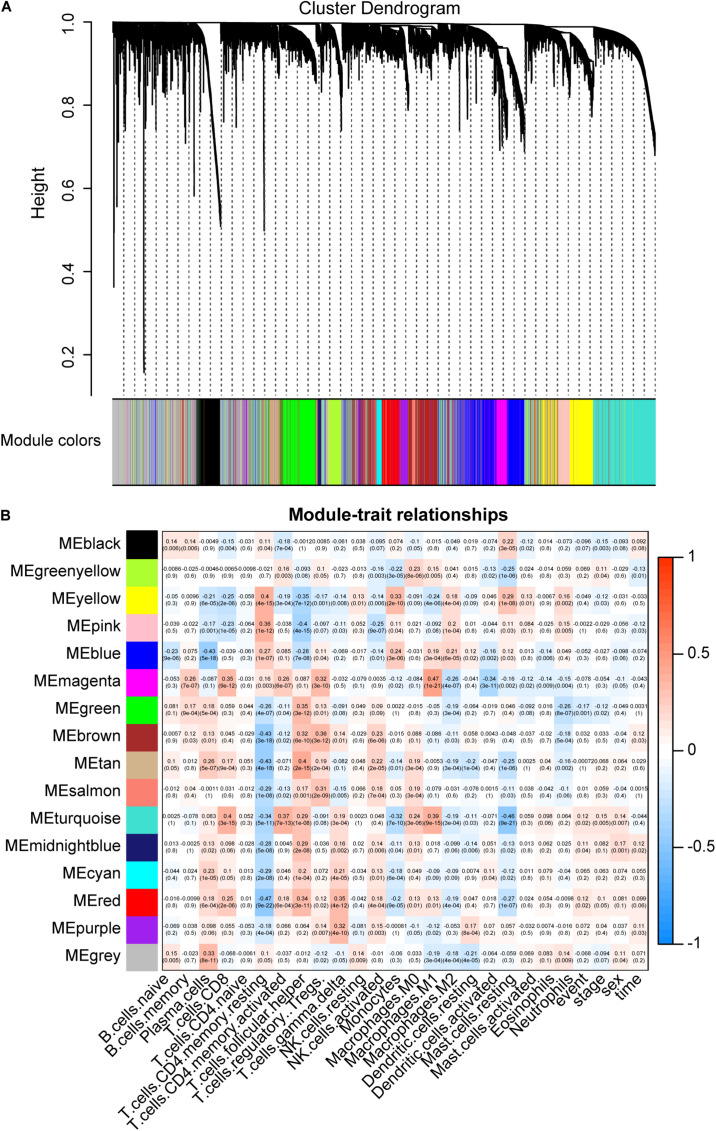
Key modules and its correlation with CIBERSORT immune fractions. **(A)** Gene modules identified by hierarchical clustering. A total of 15 non-gray modules were generated. **(B)** The correlation between modules and CIBERSORT immune fractions. The turquoise module depicts the highest correlation (*r* = 0.4, *p* = 3e-15) with CD8 T cells.

We use WGCNA to explore key modules and genes related to CIBERSORT immune fraction. In order to ensure the scale-free nature of the co-expression network, a power of β = 5 was adopted as the optimal soft threshold (Supplementary Figure S1). Next, 15 non-gray modules were obtained (Figure 4A). And the turquoise module displayed the highest correlation (*r* = 0.4, *p* = 3e-15) with CD8 T cell (Figure 4B). A total of 271 hub genes pulled from the turquoise module were labeled as CD8 T cell-related gene signatures by selecting the part that satisfies gene significance > 0.2 and intramodular connectivity > 0.7.

### Establishment of Risk Assessment Signature

**FIGURE 5:**
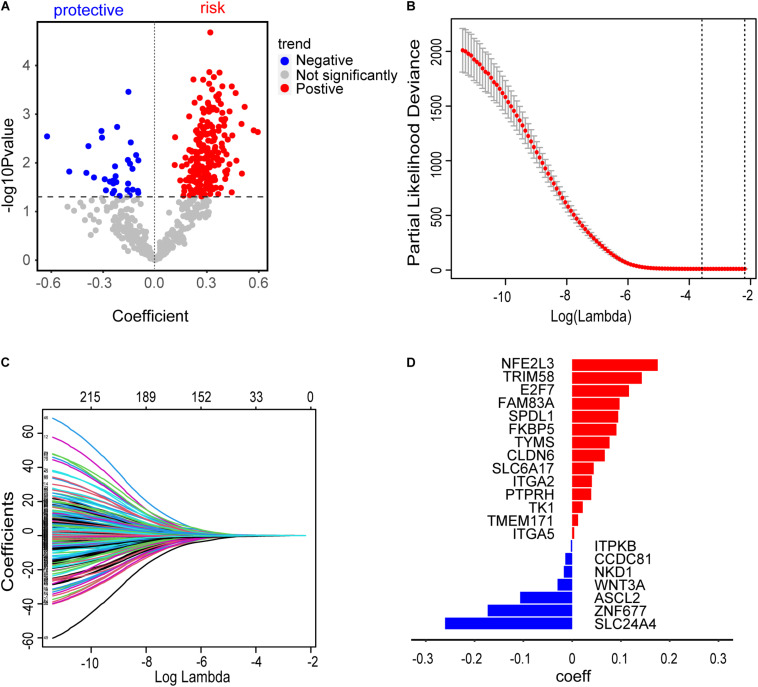
Construction of risk assessment signature. **(A)** 241 promising candidates (*P* ≤ 0.05), including 32 protective and 209 risk markers. **(B)** The optimal λ value of 0.02774519 was selected to identify the most robust markers for prognosis in LASSO regression. **(C)** A combination of 21 genes remained with their individual non-zero LASSO coefficients. **(D)** The distribution of LASSO coefficients of the gene signature. The blue bars represent protective biomarkers and the red bars represent risk biomarkers.

A total of 277 methylation driver genes and 271 CD8 T cell-related genes were integrated into a gene set. These genes were submitted to univariate Cox regression analysis. 241 potential candidate genes were pinpointed (*P* ≤ 0.05), including 209 risk and 32 protective markers (Figure 5A). In LASSO regression, the optimal λ value of 0.02774519 was adopted to point the most robust prognosis signatures, following 10-fold cross-validation with 1000 repeats (Figure 5B). The remaining 21 genes had non-zero LASSO coefficients, including SPDL1, E2F7, TK1, TYMS, ZNF677, FAM83A, TRIM58, CLDN6, NKD1, NFE2L3, FKBP5, ITGA5, ASCL2, SLC24A4, WNT3A, TMEM171, PTPRH, ITPKB, ITGA2, SLC6A17, and CCDC81 (Figure 5C). Their corresponding LASSO coefficients are shown in Figure 5D (Supplementary Table S4). Finally, the risk score was calculated according to the formula in Section “Construction of Risk Assessment Signature.”

**FIGURE 6:**
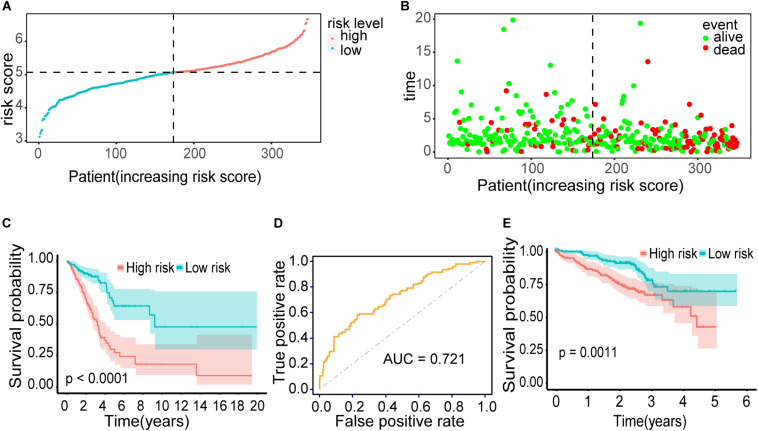
Outcomes of the prediction model. **(A)** The distribution of risk scores for 345 early-stage LUAD patients. **(B)** The distribution of survival status, sorted by risk score. **(C)** Kaplan–Meier survival curve. It demonstrated the survival difference between the high-risk group and low-risk group. **(D)** ROC curve. It showed the performance of the model. **(E)** Kaplan–Meier survival curve using the validation cohort. Patients with higher risk score exhibited worse overall survival.

The distribution of risk scores and their corresponding survival status are shown in Figures 6A,B. There was remarkable difference in survival rate between low-risk group and high-risk group (*P* = 1.933303e-09) (Figure 6C). The AUC value of the prediction model was 0.721, which revealed a worthy predictive power for the survival risk of patients with early-stage LUAD (Figure 6D). To confirm the prognostic robustness of the 21 gene signatures, it was further confirmed in an independent external cohort. Similarly, the OS rate of patients with higher risk scores is significantly worse than that of patients with lower risk scores (*p* = 0.0011, Figure 6E).

### Construction of Prognostic Nomogram

**FIGURE 7:**
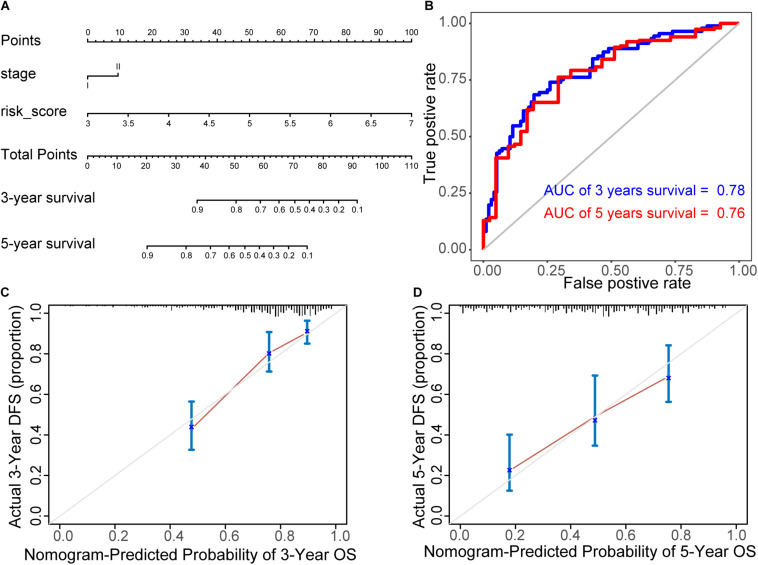
Construction of prognostic nomogram. **(A)** Nomogram for predicting 3- and 5-year survival probability, by integrating 21 biomarkers, age, risk score, sex, and tumor stage. **(B)** ROC curves of the nomogram. In the time-dependent ROC curve, the nomogram also displayed a robust performance in predicting the 3- and 5-year survival rates. The AUC was 0.78 and 0.76, respectively. **(C,D)** The calibration curves of 3- and 5-year survival rates. They showed an optimal agreement between the nomogram predictions and actual observations.

A nomogram was established to predict the 3- and 5-year survival probability, by integrating 21 biomarkers, age, risk score, sex, and tumor stage. In the univariable Cox analysis, only risk score and tumor stage were determined as independent elements (*P* < 0.01). It was further confirmed that the risk score and tumor stage were significantly related to the prognosis in multivariable Cox analysis (*P* ≤ 0.01). Thus, the prognostic nomogram was constructed by integrating these two factors (Figure 7A). In the time-dependent ROC curve, the nomogram also displayed a robust performance in predicting the 3- and 5-year survival rates. The AUC was 0.78 and 0.76, respectively (Figure 7B). The calibration curves of 3- and 5-year survival rates showed an optimal agreement between the nomogram predictions and actual observations (Figures 7C,D).

### The Further Prognostic Value of Candidate Omics Genes

A total of 141 CD8 T cell-related genes and 18 methylation driver genes were significantly associated with prognostic (*P* ≤ 0.05; Supplementary Table S5). The top six CD8 T cell-related genes were ANLN, CYP4B1, R3HDM1, KIF14, CENPK, and HJURP (Supplementary Figure S2). Patients with high expression of CYP4B1 had a higher survival rate. The top three methylation driver genes were BZW2, SLC16A3, and NKD1 (Supplementary Figure S2). Patients with hypomethylation and high expression of NKD1 had a higher survival rate.

### Drug Target TYMS

**FIGURE 8:**
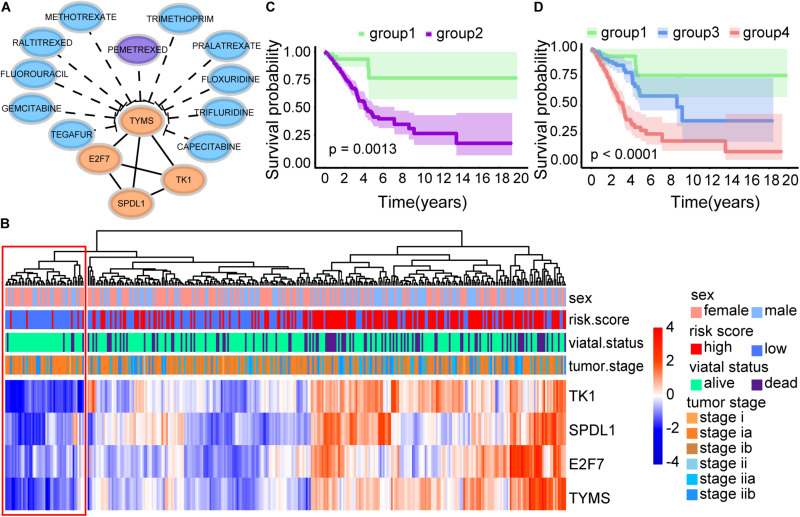
Survival analysis of four immune-related biomarkers. **(A)** Interaction network of TYMS, E2F7, SPDL1, and TK1. TYMS is the target of the FDA-approved drug Pemetrexed. **(B)** Heatmap of CD8 T cell-related genes. **(C)** Kaplan–Meier survival curve. It showed the significant survival difference between patients with low expression of TYMS, E2F7, SPDL1, TK1 (group 1), and other patients (group 2). **(D)** Kaplan–Meier survival curve. Group 2 was further classified into group 3 and group 4 according to our defined risk score index. It displayed the significant survival difference among the three groups, including the low proliferation group (group 1), the high proliferation and low risk score group (group 3), and the high proliferation and high risk score group (group 4).

Interestingly, TYMS is the target of the drug Pemetrexed according to the GDSC database (Yang et al., 2013) among 21 prognostic markers. Pemetrexed can target DNA replication by preventing both purine nucleotide and thymidine synthesis, inhibiting several folate-dependent enzymes. Three genes were interacting with TYMS among the 21 prognostic markers, which were all CD8 T cell-related genes (Figure 8A). The function of these four genes was associated with cell proliferation by GO analysis. In addition to the FDA-approved drug Pemetrexed, some potential drugs could also be used as inhibitors of TYMS according to DGIdb (Cotto et al., 2018) (Figure 8A). Patients with low expression of TYMS, E2F7, SPDL1, and TK1 showed a very high survival probability. These patients were characterized as low proliferation and termed as group 1 (Figure 8C). Further, we classified group 2 into group 3 and group 4 according to our defined risk score index. Patients with high proliferation and high-risk score group showed the worst survival status (Figure 8D).

## Discussion

The importance of methylation and immunity in tumor progression has been accepted. To date, prognostic models of LUAD were usually constructed using single-level biological factor. Focused on methylation, Kuo et al. (2016) built a prognostic panel consisting of eight signatures to predict the survival of early-stage LUAD patients in Asian and Caucasian populations. Li et al. (2019) revealed that methylation-driver mRNA and lncRNA contributed to the survival of LUAD, and eight mRNAs and four lncRNAs might be candidate biomarkers. Focused on immunity, Zhang et al. (2020) constructed the first TNF family-based model for predicting outcomes of LUAD patients, Li et al. (2017) developed and validated an individualized immune prognostic signature in early-stage non-squamous NSCLC. However, unavoidable deficiencies existed in previous studies. First, there is no study to integrate the molecular signatures of methylation and immunity into traditional prognostic systems to optimize clinical procedures in LUAD, which represents a multi-omics perspective. Second, there is no prognostic model using CD8 T cell-related genes in LUAD. Here, we used WGCNA to mine cancer biomarkers related to CD8 T cells with considering the fact that tumor infiltration is a cancer hallmark involving gene networks. Third, some patients who were clinically determined to be early stage still have a poor prognosis indicating the need for further optimization of clinical indicators. In view of the clinical complexity of LUAD, we should not directly use whole LUAD data from TCGA, but should analyze the early or late LUAD data separately.

To comprehensively explore the abnormal methylation and immune status of early-stage LUAD patients, we combined them with clinical outcomes to establish an omics prognostic index. With the consequences of LASSO COX regression analysis, the prognostic indexes based on 17 methylation driver genes (ZNF677, FAM83A, TRIM58, CLDN6, NKD1, NFE2L3, FKBP5, ITGA5, ASCL2, SLC24A4, WNT3A, TMEM171, PTPRH, ITPKB, ITGA2, SLC6A17, and CCDC81) and four CD8 T cell-related genes (SPDL1, E2F7, TK1, and TYMS) were established (Figure 5D). According to the risk score formula, each patient had a corresponding risk score. Patients with high risk value had a poorer prognosis. The survival time decreased with the increase of risk score (Figures 6B,C). We combined the risk score and the clinical pathological variables of the patient to construct a quantitative nomogram to predict the survival probability in early-stage LUAD patients (Figure 7A). Moreover, a total of 141 CD8 T cell-related genes and 18 methylation driver genes were significantly associated with prognosis. These signatures could be labeled as independent prognostic factors. Finally, we confirmed that this prognosis model could make stable predictions using GEO dataset for external verification.

Some genes involved in our study had been verified in previous studies. Zhang et al. (2019) showed that the abnormal overexpression of FAM83A in LUAD indicated a poor prognosis. Xu et al. (2017) discovered that WNT3A could regulate EMT-related proteins and promote the migration and invasion of LUAD, and it could be used as an independent prognostic factor. Malvi et al. (2019) found that TK1 was utilized as a potential target for LUAD treatment, due to its momentous role in maintaining lung tumor growth and metastasis. Shimokawa et al. (2011) analyzed the expression of thymidylate synthase (TYMS) in primary LUAD by immunohistochemistry and revealed that the high expression of TYMS might be a useful marker for predicting the recurrence of LUAD after surgery. Among the 21 prognostic markers, except for FAM83A, WNT3A, TK1, and TYMS, none of them have been reported as biomarkers in LUAD, and the function and mechanism of these genes in LUAD really need further study.

We not only analyzed 543 candidate genes, including 277 methylation driver genes and 271 CD8 T cell-related genes, but also used these two types of information to construct prognostic models separately. First, 277 methylation driver genes were submitted to univariate Cox regression analysis and LASSO COX regression model. An ensemble of 20 genes (ZNF677, FAM83A, TRIM58, RRM2, CLDN6, NKD1, NFE2L3, FKBP5, ITGA5, HMGA2, ASCL2, SLC24A4, WNT3A, TMEM171, HOXA7, PTPRH, ITPKB, ITGA2, SLC6A17, and CCDC81) was used to establish the risk score with their individual non-zero LASSO coefficients. Patients between the low-risk group and the high-risk group showed significant differences in prognosis (*P* = 5.46e-8). The AUC value of the prediction model was 0.719. Second, we performed the same process as described above on 271 CD8 T cell-related genes. A total of eight genes (CYP4B1, ECT2, PAICS, CENPU, SPDL1, CTSV, E2F7, TK1) remained with their individual non-zero LASSO coefficients. Patients between the low-risk group and the high-risk group also showed significant differences in prognosis (*P* = 0.00176501). The AUC value was 0.634. In short, the prognostic model using two biological factors had the highest AUC value, that is, 0.721. It is one of the reasons why we tried to use both 277 methylation driver genes and 271 CD8 T cell-related genes to construct a prognostic model.

Pemetrexed is a new antifolate drug that can inhibit the growth of a variety of tumors by targeting multiple folate-dependent enzymes, such as TYMS (Shih et al., 1997). In our results, E2F7, TK1, and SPDL1 were interacting with TYMS among the 21 prognostic markers (Figure 8A). And the expression trends of these four genes exhibited consistency (Figure 8B). Interestingly, patients with low expression of TYMS, E2F7, SPDL1, and TK1 showed a surprisingly high survival rate (Figure 8C). Patients with high expression of these four genes and high risk scores showed a very poor survival rate (Figure 8D). In summary, early-stage LUAD patients could be divided into three groups, including low proliferation group (group 1), high proliferation and low risk score group (group 3), and high proliferation and high risk score group (group 4) (Figure 8D). It had been suggested that high expression of TYMS in various tumor types was associated with adverse reactions to TYMS targeted drugs (Johnston et al., 1997; Pestalozzi et al., 1997). Takezawa et al. (2011) found that the expression of TYMS could be adopted as a potential predictor of the response of NSCLC patients to pemetrexed chemotherapy, because the high expression of TYMS would reduce sensitivity to pemetrexed. Our study provided a potential classification method for personalized medicine of early-stage LUAD patients. Patients in group 3, with low expression of TYMS and high risk scores, may be more suitable for pemetrexed chemotherapy.

Although the novel epigenetic and immune-related signatures and their corresponding prognostic model in this study are promising, some drawbacks still existed. First, transcriptome analysis can only reflect certain aspects of the status of CD8 T cells, but cannot reflect overall changes. Second, although the significance of these genes is indubitable in early-stage LUAD, the underlying mechanism is still unclear. Last but not least, despite the external cohort verification, the lack of experimental verification is still the main flaw of our research. Maybe we would conduct some experimental studies in the future to assist in verifying the predictions obtained from bioinformatics analysis.

## Conclusion

We utilized univariate Cox proportional hazards analysis and LASSO COX regression analysis to screen both 277 methylation driver genes and 271 CD8 T cell-related genes associated with prognosis. A prediction model was established using 17 methylation driver genes and four CD8 T cell-related genes. A nomogram combining risk score and clinicopathological factors can intuitively predict survival probability. Our study provides a robust model and candidate biomarkers for personalized therapy of early-stage LUAD patients.

## Data Availability Statement

The datasets generated during the current study are available in TCGA (https://portal.gdc.cancer.gov/) and GEO (https://www.ncbi.nlm.nih.gov/geo/) database.

## Author Contributions

MZ, XQ, and RH designed and supervised the study. JR, MZ, YY, and CL performed all the bioinformatic analyses. MZ, XQ, and RH drafted the manuscript with inputs from all other authors. All authors read and approved the final manuscript.

## Conflict of Interest

The authors declare that the research was conducted in the absence of any commercial or financial relationships that could be construed as a potential conflict of interest.

## Abbreviations

AUC: area under curve
FC: fold change
FDA: Food and Drug Administration
GDC: Genomic Data Commons
GDSC: Genomics of Drug Sensitivity in Cancer
GO: Gene Ontology
KEGG: Kyoto Encyclopedia of Genes and Genomes
LASSO: least absolute shrinkage and selection operator
LUAD: lung adenocarcinoma
NSCLC: non-small-cell lung cancer
OS: overall survival
ROC: receiver operating characteristic
SCLC: small-cell lung cancer
TCGA: The Cancer Genome Atlas
WGCNA: weighted correlation network analysis.

## References

[B1] Azimi F. Scolyer R. A. Rumcheva P. Moncrieff M. Murali R. McCarthy S. W. (2012). Tumor-infiltrating lymphocyte grade is an independent predictor of sentinel lymph node status and survival in patients with cutaneous melanoma. *J. Clin. Oncol.* 30 2678–2683. 10.1200/jco.2011.37.8539 22711850

[B2] Bernstein B. E. Meissner A. Lander E. S. (2007). The mammalian epigenome. *Cell* 128 669–681. 10.1016/j.cell.2007.01.033 17320505

[B3] Blanche P. Dartigues J. F. Jacqmin-Gadda H. (2013). Estimating and comparing time-dependent areas under receiver operating characteristic curves for censored event times with competing risks. *Stat. Med.* 32 5381–5397. 24027076 10.1002/sim.5958

[B4] Bos R. Sherman L. A. (2010). CD4+ T-cell help in the tumor milieu is required for recruitment and cytolytic function of CD8+ T lymphocytes. *Cancer Res.* 70 8368–8377. 10.1158/0008-5472.can-10-1322 20940398 PMC2970736

[B5] Cotto K. C. Wagner A. H. Feng Y.-Y. Kiwala S. Coffman A. C. Spies G. (2018). DGIdb 3.0: a redesign and expansion of the drug-gene interaction database. *Nucleic Acids Res.* 46 D1068–D1073. 29156001 10.1093/nar/gkx1143 PMC5888642

[B6] Eroglu Z. Zaretsky J. M. Hu-Lieskovan S. Kim D. W. Algazi A. Johnson D. B. (2018). High response rate to PD-1 blockade in desmoplastic melanomas. *Nature* 553 347–350. 10.1038/nature25187 29320474 PMC5773412

[B7] Friedman J. Hastie T. Tibshirani R. (2010). Regularization paths for generalized linear models via coordinate descent. *J. Stat. Softw.* 33 1–22. 20808728 PMC2929880

[B8] Galon J. Costes A. Sanchez-Cabo F. Kirilovsky A. Mlecnik B. Lagorce-Pagès C. (2006). Type, density, and location of immune cells within human colorectal tumors predict clinical outcome. *Science* 313 1960–1964. 10.1126/science.1129139 17008531

[B9] Harrell F. E. Jr. (2016). *rms: Regression modeling Strategies. R Package Version, 5.*

[B10] Herbst R. S. Heymach J. V. Lippman S. M. (2008). Lung cancer. *N. Engl. J. Med.* 359 1367–1380. 18815398 10.1056/NEJMra0802714 PMC10662965

[B11] Herbst R. S. Soria J.-C. Kowanetz M. Fine G. D. Hamid O. Gordon M. S. (2014). Predictive correlates of response to the anti-PD-L1 antibody MPDL3280A in cancer patients. *Nature* 515 563–567. 25428504 10.1038/nature14011 PMC4836193

[B12] Hiraoka K. Miyamoto M. Cho Y. Suzuoki M. Oshikiri T. Nakakubo Y. (2006). Concurrent infiltration by CD8+ T cells and CD4+ T cells is a favourable prognostic factor in non-small-cell lung carcinoma. *Br. J. Cancer* 94 275–280. 10.1038/sj.bjc.6602934 16421594 PMC2361103

[B13] Johnston P. G. Mick R. Recant W. Behan K. A. Dolan M. E. Ratain M. J. (1997). Thymidylate synthase expression and response to neoadjuvant chemotherapy in patients with advanced head and neck cancer. *J. Natl. Cancer Inst.* 89 308–313. 10.1093/jnci/89.4.308 9048835

[B14] Kuo I. Y. Jen J. Hsu L.-H. Hsu H.-S. Lai W.-W. Wang Y.-C. (2016). A prognostic predictor panel with DNA methylation biomarkers for early-stage lung adenocarcinoma in Asian and Caucasian populations. *J. Biomed. Sci.* 23:58. 10.1186/s12929-016-0276-x PMC4969679 27484806

[B15] Langfelder P. Horvath S. (2008). WGCNA: an R package for weighted correlation network analysis. *BMC Bioinformatics* 9:559. 10.1186/1471-2105-9-559 19114008 PMC2631488

[B16] Li B. Cui Y. Diehn M. Li R. (2017). Development and validation of an individualized immune prognostic signature in early-stage nonsquamous non-small cell Lung Cancer. *JAMA Oncol.* 3 1529–1537. 10.1001/jamaoncol.2017.1609 28687838 PMC5710196

[B17] Li R. Yang Y.-E. Yin Y.-H. Zhang M.-Y. Li H. Qu Y.-Q. (2019). Methylation and transcriptome analysis reveal lung adenocarcinoma-specific diagnostic biomarkers. *J. Transl. Med.* 17:324. 10.1186/s12967-019-2068-z PMC6764142 31558162

[B18] Love M. I. Huber W. Anders S. (2014). Moderated estimation of fold change and dispersion for RNA-seq data with DESeq2. *Genome Biol.* 15:550. 10.1186/s13059-014-0550-8 PMC4302049 25516281

[B19] Malvi P. Janostiak R. Nagarajan A. Cai G. Wajapeyee N. (2019). Loss of thymidine kinase 1 inhibits lung cancer growth and metastatic attributes by reducing GDF15 expression. *PLoS Genet.* 15:e1008439. 10.1371/journal.pgen.1008439 31589613 PMC6797230

[B20] Muller D. C. Larose T. L. Hodge A. Guida F. Langhammer A. Grankvist K. (2019). Circulating high sensitivity C reactive protein concentrations and risk of lung cancer: nested case-control study within Lung Cancer Cohort Consortium. *Bmj* 364:k4981. 10.1136/bmj.k4981 PMC6315896 30606716

[B21] Nakanishi Y. Lu B. Gerard C. Iwasaki A. (2009). CD8(+) T lymphocyte mobilization to virus-infected tissue requires CD4(+) T-cell help. *Nature* 462 510–513. 10.1038/nature08511 19898495 PMC2789415

[B22] Newman A. M. Liu C. L. Green M. R. Gentles A. J. Feng W. Xu Y. (2015). Robust enumeration of cell subsets from tissue expression profiles. *Nat. Methods* 12 453–457. 10.1038/nmeth.3337 25822800 PMC4739640

[B23] Pagès F. Berger A. Camus M. Sanchez-Cabo F. Costes A. Molidor R. (2005). Effector memory T cells, early metastasis, and survival in colorectal cancer. *N. Engl. J. Med.* 353 2654–2666. 10.1056/nejmoa051424 16371631

[B24] Peranzoni E. Lemoine J. Vimeux L. Feuillet V. Barrin S. Kantari-Mimoun C. (2018). Macrophages impede CD8 T cells from reaching tumor cells and limit the efficacy of anti-PD-1 treatment. *Proc. Natl. Acad. Sci. U.S.A.* 115 E4041–E4050. 29632196 10.1073/pnas.1720948115 PMC5924916

[B25] Pestalozzi B. C. Peterson H. F. Gelber R. D. Goldhirsch A. Gusterson B. A. Trihia H. (1997). Prognostic importance of thymidylate synthase expression in early breast cancer. *J. Clin. Oncol.* 15 1923–1931. 9164203 10.1200/JCO.1997.15.5.1923

[B26] Pignon J.-P. Tribodet H. Scagliotti G. V. Douillard J.-Y. Shepherd F. A. Stephens R. J. (2008). Lung adjuvant cisplatin evaluation: a pooled analysis by the LACE Collaborative Group. *J. Clin. Oncol.* 26 3552–3559. 10.1200/jco.2007.13.9030 18506026

[B27] Savas P. Virassamy B. Ye C. Salim A. Mintoff C. P. Caramia F. (2018). Single-cell profiling of breast cancer T cells reveals a tissue-resident memory subset associated with improved prognosis. *Nat. Med.* 24 986–993. 10.1038/s41591-018-0078-7 29942092

[B28] Shih C. Chen V. J. Gossett L. S. Gates S. B. MacKellar W. C. Habeck L. L. (1997). LY231514, a pyrrolo[2,3-d]pyrimidine-based antifolate that inhibits multiple folate-requiring enzymes. *Cancer Res.* 57 1116–1123. 9067281

[B29] Shimokawa H. Uramoto H. Onitsuka T. Iwata T. Nakagawa M. Ono K. (2011). TS expression predicts postoperative recurrence in adenocarcinoma of the lung. *Lung Cancer* 72 360–364. 10.1016/j.lungcan.2010.08.024 20970877

[B30] Sing T. Sander O. Beerenwinkel N. Lengauer T. (2005). ROCR: visualizing classifier performance in R. *Bioinformatics* 21 3940–3941. 10.1093/bioinformatics/bti623 16096348

[B31] Strauss G. M. Herndon J. E. Maddaus M. A. Johnstone D. W. Johnson E. A. Harpole D. H. (2008). Adjuvant paclitaxel plus carboplatin compared with observation in stage IB non-small-cell lung cancer: CALGB 9633 with the Cancer and Leukemia Group B, Radiation Therapy Oncology Group, and North Central Cancer Treatment Group Study Groups. *J. Clin. Oncol.* 26 5043–5051. 10.1200/jco.2008.16.4855 18809614 PMC2652093

[B32] Takezawa K. Okamoto I. Okamoto W. Takeda M. Sakai K. Tsukioka S. (2011). Thymidylate synthase as a determinant of pemetrexed sensitivity in non-small cell lung cancer. *Br. J. Cancer* 104 1594–1601. 10.1038/bjc.2011.129 21487406 PMC3101907

[B33] Tian Y. Morris T. J. Webster A. P. Yang Z. Beck S. Feber A. (2017). ChAMP: updated methylation analysis pipeline for Illumina BeadChips. *Bioinformatics* 33 3982–3984. 10.1093/bioinformatics/btx513 28961746 PMC5860089

[B34] Tomczak K. Czerwiñska P. Wiznerowicz M. (2015). The cancer genome atlas (TCGA): an immeasurable source of knowledge. *Contemp. Oncol.* 19 A68–A77. 10.5114/wo.2014.47136 PMC4322527 25691825

[B35] Tumeh P. C. Harview C. L. Yearley J. H. Shintaku I. P. Taylor E. J. M. Robert L. (2014). PD-1 blockade induces responses by inhibiting adaptive immune resistance. *Nature* 515 568–571. 25428505 10.1038/nature13954 PMC4246418

[B36] Xu J. Lv W. Hu Y. Wang L. Wang Y. Cao J. (2017). Wnt3a expression is associated with epithelial-mesenchymal transition and impacts prognosis of lung adenocarcinoma patients. *J. Cancer* 8 2523–2531. 10.7150/jca.18560 28900490 PMC5595082

[B37] Yang W. Soares J. Greninger P. Edelman E. J. Lightfoot H. Forbes S. (2013). Genomics of Drug Sensitivity in Cancer (GDSC): a resource for therapeutic biomarker discovery in cancer cells. *Nucleic Acids Res.* 41 D955–D961. 23180760 10.1093/nar/gks1111 PMC3531057

[B38] Yang Z. Liu B. Lin T. Zhang Y. Zhang L. Wang M. (2019). Multiomics analysis on DNA methylation and the expression of both messenger RNA and microRNA in lung adenocarcinoma. *J. Cell Physiol.* 234 7579–7586. 10.1002/jcp.27520 30370535

[B39] Yu G. Wang L.-G. Han Y. He Q.-Y. (2012). clusterProfiler: an R package for comparing biological themes among gene clusters. *OMICS* 16 284–287. 10.1089/omi.2011.0118 22455463 PMC3339379

[B40] Zhang C. Zhang G. Sun N. Zhang Z. Zhang Z. Luo Y. (2020). Comprehensive molecular analyses of a TNF family-based signature with regard to prognosis, immune features, and biomarkers for immunotherapy in lung adenocarcinoma. *EBioMedicine* 59:102959. 10.1016/j.ebiom.2020.102959 32853987 PMC7452643

[B41] Zhang J. Sun G. Mei X. (2019). Elevated FAM83A expression predicts poorer clincal outcome in lung adenocarcinoma. *Cancer Biomark. Sect. A Dis. Mark.* 26 367–373. 10.3233/cbm-190520 31594212 PMC12826422

[B42] Zheng X. Zhang N. Wu H.-J. Wu H. (2017). Estimating and accounting for tumor purity in the analysis of DNA methylation data from cancer studies. *Genome Biol.* 18:17. 10.1186/s13059-016-1143-5 PMC5267453 28122605

