# Supplementary figures and images for: A Novel Prognostic Model of Early-Stage Lung Adenocarcinoma Integrating Methylation and Immune Biomarkers
Source: Front Genet. 2021 Jan 21;11:634634. doi: 10.3389/fgene.2020.634634 (PMC7859522; doi:10.3389/fgene.2020.634634)

# Scale independence

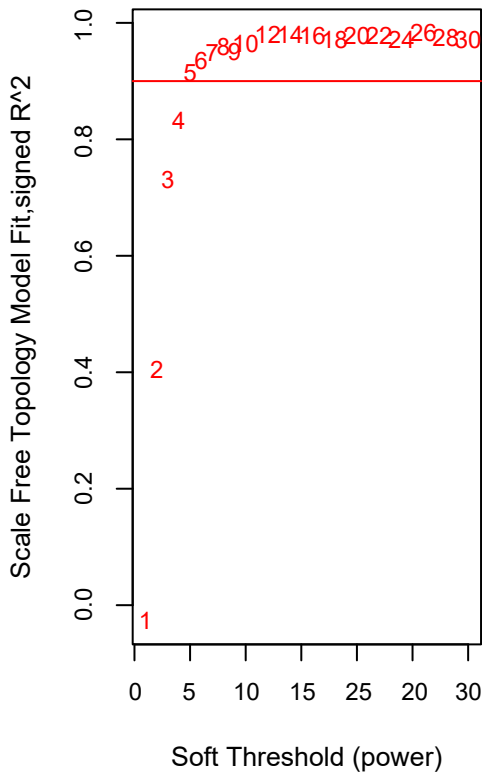

# Mean connectivity

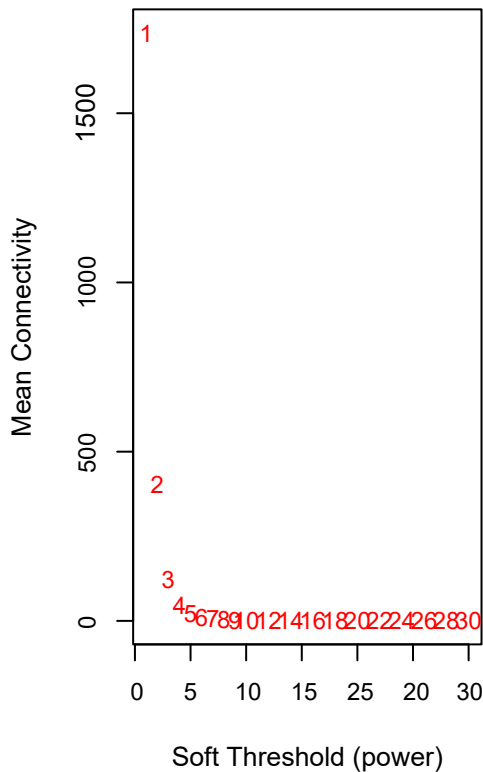

Supplement: Supplementary file 1 [file Image_1.PDF]

## The further prognostic value of candidate omics genes

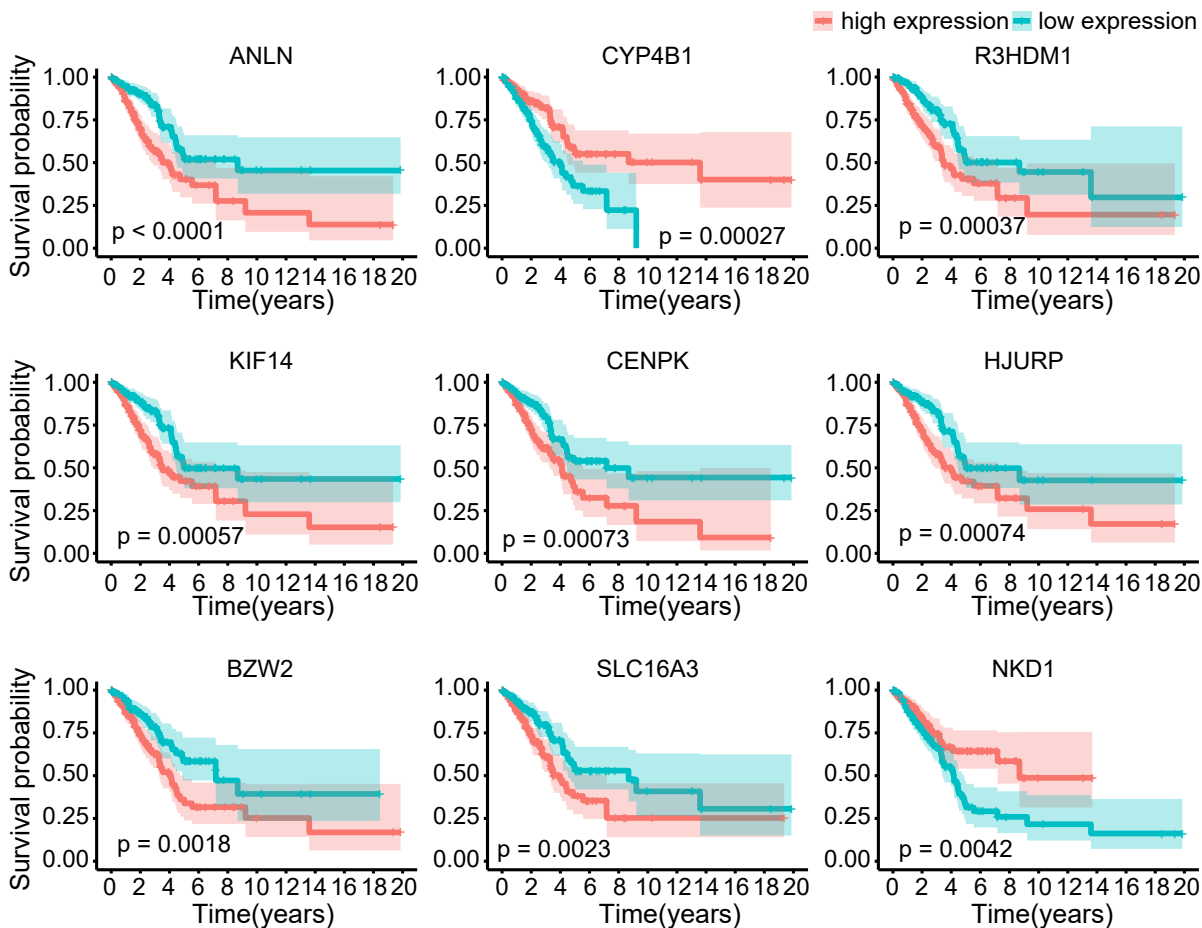

Supplement: Supplementary file 2 [file Image_2.PDF]
