# Supplementary material for: A Novel Prognostic Model of Early-Stage Lung Adenocarcinoma Integrating Methylation and Immune Biomarkers
Source: Front Genet. 2021 Jan 21;11:634634. doi: 10.3389/fgene.2020.634634 (PMC7859522; doi:10.3389/fgene.2020.634634)
Supplement: Supplementary file 3 [file Presentation_1.PDF]

Supplementary Material

1 Supplementary Figures and Tables

1.1 Supplementary Figures

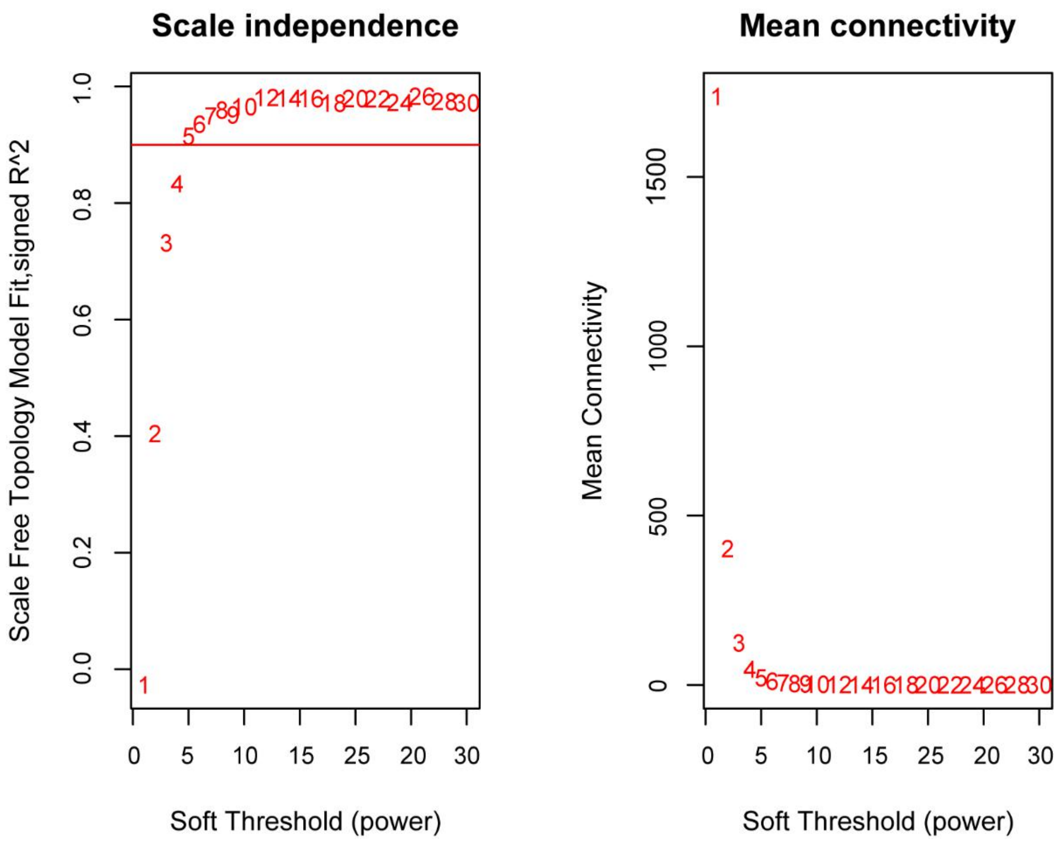

Figure S1. Soft threshold selection in the WGCNA network analysis.Obviously, the appropriate power value is 5 because here the mean connectivity is closest to zero and the power value is relatively smaller.

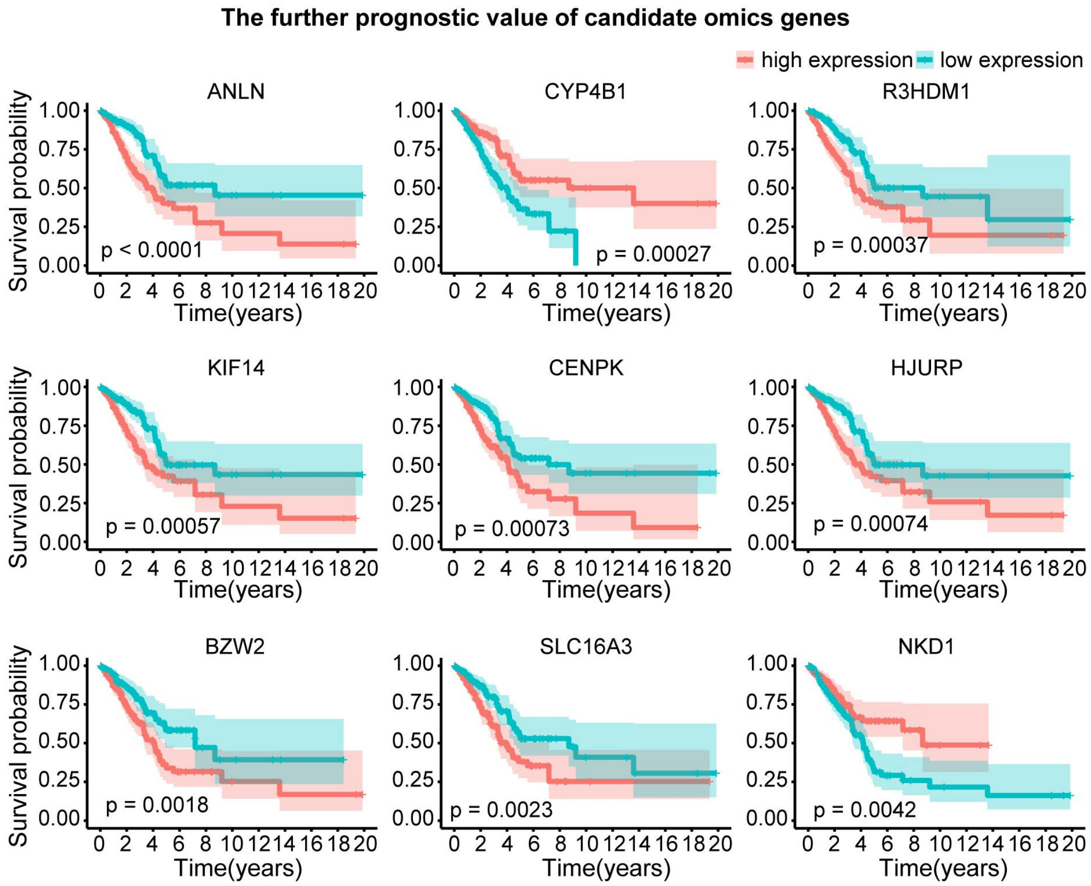

Figure S2. Top 9 genes significantly associated with prognosis. The first 6 genes were related to immune infiltration. Methylation driver genes were BZW2, SLC16A3, NKD1. Patients with high expression of CYP4B1 and NKD1 had a higher survival rate. Besides, Patients with high expression of these genes had a worse prognosis.

Supplementary Tables:

Table S1. Detailed information of 277 methylation driver genes.

Table S2. The enriched terms of the biological process of GO.

Table S3. Result of KEGG enrichment analysis.

Table S4. LASSO coefficients of 21 biomarkers.

Table S5. Genes significantly associated with prognosis in joint survival analysis
